# Supplementary material for: Combined Exposure to Birch Pollen and Thunderstorms Affects Respiratory Health in Stockholm, Sweden—A Time Series Analysis
Source: Int J Environ Res Public Health. 2022 May 11;19(10):5852. doi: 10.3390/ijerph19105852 (PMC9141405; doi:10.3390/ijerph19105852)
Supplement: Supplementary file 1 [file ijerph-19-05852-s001.zip › ijerph-1699527-supplementary.pdf]

Supplementary material

Table S1. The average number of daily outpatient visits due to respiratory causes during the study period per year (SD – Standard Deviation; Min – minimum; Max – maximum)

Year	Mean	SD	Min	Max
2002	230.4	147.7	39	545
2003	221.3	144.5	34	503
2004	233.3	149.9	36	576
2005	241.7	158.4	35	557
2006	269.2	179.8	39	793
2007	263.4	175.8	44	623
2008	254.2	170.0	36	597
2009	270.4	179.2	28	608
2010	294.1	191.9	40	645
2011	298.9	198.7	43	671
2012	331.6	230.0	40	802
2013	322.6	229.7	35	760
2014	378.3	269.5	45	867
2015	381.5	252.9	52	873
2016	410.6	263.3	58	878
2017	418.0	272.4	41	913
Total	301.2	214.6	28	913

Table S2. Full results of the Model 1a: the association between the daily number of outpatient visits due to respiratory causes, birch pollen concentration (divided into five categories) and thunder-storm-associated lightning discharges (divided into five categories) on the same day (CI – confidence interval).

Variable	β (CI 95 %)
Period trends:	
sin 24	1.03 (1.02; 1.05)
sin 12	1.17 (1.15; 1.2)
sin 6	0.89 (0.87; 0.9)
sin 4	1.1 (1.08; 1.11)
sin 3	0.99 (0.98; 1.01)
cos 24	0.99 (0.98; 1.01)
cos 12	1.52 (1.49; 1.56)
cos 6	0.84 (0.82; 0.85)
cos 4	1.04 (1.03; 1.06)
cos 3	0.99 (0.98; 1.01)
Day number	1.0002 (1.0002; 1.0003)
(Day number)^2	1 (1; 1)
Day of the week:	
Sunday	Ref.
Monday	5.71 (5.46; 5.97)
Tuesday	5.92 (5.68; 6.17)

Wednesday	5.26 (5.05; 5.48)
Thursday	4.89 (4.64; 5.16)
Friday	3.16 (3.02; 3.32)
Saturday	0.94 (0.9; 0.98)
Birch pollen:	
cat 0: 0	Ref.
cat 1: 1	1 (0.96; 1.04)
cat 2: 2-6	1.01 (0.95; 1.06)
cat 3: 17-39	1.03 (0.96; 1.1)
cat 4: 41-4759	1.05 (0.97; 1.15)
Lightning discharges (n/day):	
cat 0: 0	Ref.
cat 1: 1-13	1.01 (0.99; 1.04)
cat 2: 14-90	1.01 (0.98; 1.04)
cat 3: 91-249	0.98 (0.94; 1.02)
cat 4: ≥250	0.98 (0.95; 1.01)
Interactions:	
birch cat 1 x lightning cat 1	0.99 (0.93; 1.06)
birch cat 1 x lightning cat 2	1.07 (1; 1.13)
birch cat 1 x lightning cat 3	0.95 (0.89; 1.01)
birch cat 1 x lightning cat 4	1.1 (0.97; 1.24)
birch cat 2 x lightning cat 1	1 (0.91; 1.1)
birch cat 2 x lightning cat 2	0.99 (0.82; 1.19)
birch cat 2 x lightning cat 3	1.07 (0.97; 1.18)
birch cat 2 x lightning cat 4	1.13 (1.04; 1.23)
birch cat 3 x lightning cat 1	1.07 (1.01; 1.14)
birch cat 3 x lightning cat 2	0.89 (0.68; 1.16)
birch cat 3 x lightning cat 3	0.99 (0.92; 1.07)
birch cat 3 x lightning cat 4	1.07 (1; 1.14)
birch cat 4 x lightning cat 1	0.93 (0.81; 1.07)
birch cat 4 x lightning cat 2	1.06 (0.95; 1.17)
birch cat 4 x lightning cat 3	1.19 (1.08; 1.31)
birch cat 4 x lightning cat 4	1.19 (1.11; 1.28)
Temperature (linear spline with one knot at 12° C):	
temp1	1.001 (0.992; 1.01)
temp2	1 (omitted)
Relative Humidity (Restricted Cubic Spline):	
RH1	1.001 (0.998; 1.004)
RH2	0.998 (0.996; 1.001)
Wind speed	1.027 (1.013; 1.042)
Air pressure	1 (0.998; 1.001)
NO_x	1.006 (1.004; 1.008)
Intercept	63.94 (19.26; 212.3)

Table S3. Full results of the Model 2a, which investigated the association between the number of outpatient visits, and the exposure variables – birch pollen concentration and the number of lightning discharges – on day before the outpatient visit (CI – confidence interval).

Variable	β (CI 95 %)
Period trends:	
sin 24	1.02 (1; 1.04)
sin 12	1.15 (1.12; 1.17)
sin 6	0.86 (0.85; 0.88)
sin 4	1.08 (1.06; 1.1)
sin 3	0.98 (0.97; 0.99)
cos 24	0.99 (0.98; 1)
cos 12	1.53 (1.49; 1.56)
cos 6	0.83 (0.82; 0.85)
cos 4	1.05 (1.04; 1.06)
cos 3	1 (0.99; 1.02)
Day number	1.0002 (1.0001; 1.0003)
(Day number)^2	1 (1; 1)
Day of the week:	
Sunday	Ref.
Monday	6.04 (5.81; 6.29)
Tuesday	6.16 (5.93; 6.4)
Wednesday	5.54 (5.34; 5.74)
Thursday	5.13 (4.9; 5.38)
Friday	3.25 (3.1; 3.41)
Saturday	0.93 (0.89; 0.98)
Birch pollen:	
cat 0: 0	Ref.
cat 1: 1	1.04 (1; 1.07)
cat 2: 2-6	1.05 (0.99; 1.11)
cat 3: 17-39	1.11 (1.03; 1.19)
cat 4: 41-4759	1.19 (1.1; 1.29)
Lightning discharges (n/day):	
cat 0: 0	Ref.
cat 1: 1-13	1.01 (0.99; 1.04)
cat 2: 14-90	0.97 (0.92; 1.03)
cat 3: 91-249	1.01 (0.97; 1.05)
cat 4: ≥250	1.02 (0.98; 1.07)
Interactions:	
birch cat 1 x lightning cat 1	1.02 (0.95; 1.09)
birch cat 1 x lightning cat 2	1.04 (0.96; 1.13)
birch cat 1 x lightning cat 3	0.91 (0.86; 0.98)
birch cat 1 x lightning cat 4	0.96 (0.87; 1.06)
birch cat 2 x lightning cat 1	0.93 (0.77; 1.12)
birch cat 2 x lightning cat 2	0.97 (0.74; 1.27)
birch cat 2 x lightning cat 3	1.02 (0.91; 1.14)
birch cat 2 x lightning cat 4	1.05 (0.96; 1.14)
birch cat 3 x lightning cat 1	0.95 (0.82; 1.11)

birch cat 3 x lightning cat 2	1.05 (0.93; 1.18)
birch cat 3 x lightning cat 3	0.92 (0.75; 1.14)
birch cat 3 x lightning cat 4	1.03 (0.97; 1.1)
birch cat 4 x lightning cat 1	0.8 (0.66; 0.98)
birch cat 4 x lightning cat 2	1.06 (0.97; 1.15)
birch cat 4 x lightning cat 3	1.16 (1.05; 1.27)
birch cat 4 x lightning cat 4	1.08 (1.01; 1.17)
Temperature (linear spline with one knot at 12° C):	
temp1	1.002 (0.994; 1.01)
temp2	1 (omitted)
Relative Humidity (Restricted Cubic Spline):	
RH1	1.003 (0.999; 1.006)
RH2	0.998 (0.995; 1.001)
Wind speed	1 (0.986; 1.014)
Air pressure	1 (0.998; 1.001)
NO_x	1 (0.999; 1.002)
Intercept	48.42 (12.2; 192.09)

Table S4. Full results of the Model 2a, which investigated the association between the number of outpatient visits, and the exposure variables – birch pollen concentration and the number of lightning discharges – on day before the outpatient visit (CI – confidence interval).

Variable	β (CI 95 %)
Study month:	
1 (May 2002)	Ref.
2 (June 2002)	0.98 (0.84; 1.15)
3 (July 2002)	0.54 (0.46; 0.64)
4 (August 2002)	0.82 (0.68; 0.99)
5 (September 2002)	1.12 (0.95; 1.32)
6 (May 2003)	0.94 (0.79; 1.12)
7 (June 2003)	0.92 (0.76; 1.12)
8 (July 2003)	0.54 (0.45; 0.64)
9 (August 2003)	0.81 (0.66; 0.99)
10 (September 2003)	1.16 (0.98; 1.37)
11 (May 2004)	1.03 (0.86; 1.22)
12 (June 2004)	1.03 (0.87; 1.23)
13 (July 2004)	0.5 (0.42; 0.6)
14 (August 2004)	0.86 (0.7; 1.06)
15 (September 2004)	1.2 (1.02; 1.41)
16 (May 2005)	1.12 (0.97; 1.3)
17 (June 2005)	1.06 (0.87; 1.28)
18 (July 2005)	0.54 (0.44; 0.65)
19 (August 2005)	0.8 (0.65; 0.98)
20 (September 2005)	1.24 (1.05; 1.46)
21 (May 2006)	1.15 (0.93; 1.44)
22 (June 2006)	1.09 (0.89; 1.34)

23 (July 2006)	0.61 (0.49; 0.75)
24 (August 2006)	0.85 (0.7; 1.05)
25 (September 2006)	1.28 (1.08; 1.51)
26 (May 2007)	1.26 (1.07; 1.49)
27 (June 2007)	1.14 (0.94; 1.37)
28 (July 2007)	0.61 (0.5; 0.73)
29 (August 2007)	0.95 (0.78; 1.16)
30 (September 2007)	1.34 (1.13; 1.58)
31 (May 2008)	1.19 (1.02; 1.4)
32 (June 2008)	1.05 (0.9; 1.23)
33 (July 2008)	0.6 (0.5; 0.73)
34 (August 2008)	0.85 (0.69; 1.06)
35 (September 2008)	1.35 (1.14; 1.59)
36 (May 2009)	1.19 (1.02; 1.39)
37 (June 2009)	1.19 (1.01; 1.41)
38 (July 2009)	0.6 (0.5; 0.73)
39 (August 2009)	1.03 (0.84; 1.26)
40 (September 2009)	1.45 (1.23; 1.71)
41 (May 2010)	1.26 (1.08; 1.46)
42 (June 2010)	1.28 (1.1; 1.49)
43 (July 2010)	0.66 (0.54; 0.81)
44 (August 2010)	1.02 (0.82; 1.27)
45 (September 2010)	1.54 (1.31; 1.81)
46 (May 2011)	1.46 (1.26; 1.69)
47 (June 2011)	1.25 (1.01; 1.55)
48 (July 2011)	0.65 (0.54; 0.78)
49 (August 2011)	1.04 (0.84; 1.29)
50 (September 2011)	1.57 (1.33; 1.84)
51 (May 2012)	1.48 (1.21; 1.82)
52 (June 2012)	1.49 (1.23; 1.81)
53 (July 2012)	0.76 (0.62; 0.92)
54 (August 2012)	1.24 (1.02; 1.51)
55 (September 2012)	1.59 (1.33; 1.89)
56 (May 2013)	1.69 (1.43; 2)
57 (June 2013)	1.6 (1.32; 1.94)
58 (July 2013)	0.73 (0.6; 0.9)
59 (August 2013)	1.23 (1.01; 1.5)
60 (September 2013)	1.64 (1.39; 1.94)
61 (May 2014)	1.58 (1.34; 1.86)
62 (June 2014)	1.69 (1.44; 1.99)
63 (July 2014)	0.87 (0.71; 1.06)
64 (August 2014)	1.46 (1.19; 1.78)
65 (September 2014)	2.01 (1.71; 2.38)
66 (May 2015)	1.71 (1.45; 2.02)
67 (June 2015)	1.79 (1.52; 2.12)

68 (July 2015)	0.87 (0.71; 1.06)
69 (August 2015)	1.45 (1.19; 1.76)
70 (September 2015)	1.91 (1.62; 2.25)
71 (May 2016)	1.73 (1.48; 2.03)
72 (June 2016)	1.73 (1.39; 2.14)
73 (July 2016)	0.99 (0.82; 1.2)
74 (August 2016)	1.5 (1.23; 1.83)
75 (September 2016)	2.12 (1.81; 2.49)
76 (May 2017)	1.78 (1.51; 2.11)
77 (June 2017)	1.82 (1.52; 2.19)
78 (July 2017)	0.99 (0.82; 1.19)
79 (August 2017)	1.55 (1.28; 1.9)
80 (September 2017)	2.11 (1.79; 2.49)
Day of the week:	
Sunday	Ref.
Monday	6.05 (5.8; 6.31)
Tuesday	6.06 (5.81; 6.32)
Wednesday	5.44 (5.22; 5.67)
Thursday	5.03 (4.79; 5.28)
Friday	3.19 (3.03; 3.36)
Saturday	0.93 (0.88; 0.97)
Birch pollen:	
cat 0: 0	Ref.
cat 1: 1	1.04 (1; 1.09)
cat 2: 2-6	1.1 (1.03; 1.17)
cat 3: 17-39	1.23 (1.12; 1.35)
cat 4: 41-4759	1.33 (1.19; 1.48)
Lightning discharges (n/day):	
cat 0: 0	Ref.
cat 1: 1-13	1.01 (0.96; 1.06)
cat 2: 14-90	0.92 (0.85; 0.99)
cat 3: 91-249	0.99 (0.92; 1.06)
cat 4: ≥250	0.99 (0.92; 1.07)
Interactions:	
birch cat 1 x lightning cat 1	1.01 (0.92; 1.11)
birch cat 1 x lightning cat 2	1.1 (0.97; 1.24)
birch cat 1 x lightning cat 3	1.09 (0.95; 1.24)
birch cat 1 x lightning cat 4	1.07 (0.9; 1.27)
birch cat 2 x lightning cat 1	0.94 (0.8; 1.11)
birch cat 2 x lightning cat 2	1.05 (0.83; 1.33)
birch cat 2 x lightning cat 3	1.09 (0.95; 1.24)
birch cat 2 x lightning cat 4	1.07 (0.93; 1.23)
birch cat 3 x lightning cat 1	0.93 (0.8; 1.08)
birch cat 3 x lightning cat 2	1.13 (0.98; 1.3)
birch cat 3 x lightning cat 3	0.92 (0.73; 1.15)

birch cat 3 x lightning cat 4	1.1 (0.99; 1.22)
birch cat 4 x lightning cat 1	0.8 (0.64; 1)
birch cat 4 x lightning cat 2	1.1 (0.99; 1.22)
birch cat 4 x lightning cat 3	1.11 (1; 1.24)
birch cat 4 x lightning cat 4	1.06 (0.9; 1.26)
Temperature (linear spline with one knot at 12° C):	
temp1	0.995 (0.988; 1.003)
temp2	1 (omitted)
Relative Humidity (Restricted Cubic Spline):	
RH1	1.001 (0.998; 1.004)
RH2	1 (0.996; 1.003)
Wind speed	1.011 (0.995; 1.027)
Air pressure	1 (0.998; 1.001)
NO_x	1.002 (1; 1.004)
Intercept	64.25 (12.32; 335.15)
